# Personalizing Heart Rate-Based Seizure Detection Using Supervised SVM Transfer Learning

**DOI:** 10.3389/fneur.2020.00145

**Published:** 2020-02-26

**Authors:** Thomas De Cooman, Kaat Vandecasteele, Carolina Varon, Borbála Hunyadi, Evy Cleeren, Wim Van Paesschen, Sabine Van Huffel

**Affiliations:** ^1^Department of Electrical Engineering (ESAT), STADIUS Center for Dynamical Systems, Signal Processing and Data Analytics, KU Leuven, Leuven, Belgium; ^2^Department of Microelectronics, TU Delft, Delft, Netherlands; ^3^Department of Neurosciences, University Hospitals Leuven, KU Leuven, Leuven, Belgium

**Keywords:** epilepsy, transfer learning, seizure detection, personalization, heart rate analysis, SVM

## Abstract

**Objective:** Automated seizure detection is a key aspect of wearable seizure warning systems. As a result, the quality of life of refractory epilepsy patients could be improved. Most state-of-the-art algorithms for heart rate-based seizure detection use a so-called patient-independent approach, which do not take into account patient-specific data during algorithm training. Although such systems are easy to use in practice, they lead to many false detections as the ictal heart rate changes are patient-dependent. In practice, only a limited amount of accurately annotated patient data is typically available, which makes it difficult to create fully patient-specific algorithms.

**Methods:** In this context, this study proposes for the first time a new transfer learning approach that allows to personalize heart rate-based seizure detection by using only a couple of days of data per patient. The algorithm was evaluated on 2,172 h of single-lead ECG data from 24 temporal lobe epilepsy patients including 227 focal impaired awareness seizures.

**Results:** The proposed personalized approach resulted in an overall sensitivity of 71% with 1.9 false detections per hour. This is an average decrease in false detection rate of 37% compared to the reference patient-independent algorithm using only a limited amount of personal seizure data. The proposed transfer learning approach adapts faster and more robustly to patient-specific characteristics than other alternatives for personalization in the literature.

**Conclusion:** The proposed method allows an easy implementable solution to personalize heart rate-based seizure detection, which can improve the quality of life of refractory epilepsy patients when used as part of a multimodal seizure detection system.

## 1. Introduction

Epilepsy is one of the most common neurological disorders, which affects around 1% of the population worldwide (1). Anti-epileptic drugs provide adequate treatment for about 70% of epilepsy patients (2). The remaining 30% of the patients continue to have seizures, which drastically affects their quality of life. This can be improved by an automated warning system that alarms the parents or caregivers when the patient experiences a seizure. By using such a system, the patients and relatives feel more at ease knowing someone will be able to help the patient out when a seizure would occur. Quick intervention can then lead to a decrease in injuries and avoid (post-)ictal complications, including sudden unexplained death in epilepsy (SUDEP). In addition, a seizure diary, automatically generated from the alarms, can be used for a follow-up of the disease and evaluation of the treatment. A seizure diary, kept by the patients or their families, has proven to be unreliable, which leads to bad treatment follow-up (3). An automatically generated seizure diary could lead to a more objective seizure count and an improved treatment selection.

The key element of such a warning system is the automated seizure detection algorithm. In the literature, these algorithms are typically based on full electroencephalography (EEG). EEG recordings mostly require wet electrodes on the scalp, which is uncomfortable for a long-term monitoring solution (4). More easily obtainable biomedical signals used to detect epileptic seizures include accelerometry (ACC), electromyography (EMG), electrocardiography (ECG) and galvanic skin response (5). The most suitable modality or combination of modalities depends on the seizure type. ECG-based seizure detection, for instance, is ideal for the detection of focal impaired awareness seizures (FIAS) arising from the temporal lobe, as they are not accompanied by typical motor components, but they are associated with ictal tachycardia (6–8). Therefore, this study focuses on patients with temporal lobe epilepsy, as for this type of focal seizures, ECG-based seizure detection is of most added value compared to other wearable modalities. It should however be noted that ECG-based seizure detection algorithms can be used for a wider range of seizure types, including focal seizures with non-temporal seizure onsets and generalized tonic-clonic seizures (9). It is also a very important modality for long-term monitoring applications as it allows to assess the patient's general health status (e.g., sleep and general heart conditions).

Most ECG-based seizure detection systems from the literature are based on patient-independent models (10–13). For this type of models, no patient-specific data is required, making them directly usable in practice. However, due to the large inter-patient differences in heart rate characteristics, performance is too low for practical use.

In order to increase the performance, models can be adapted to the patient heart rate characteristics (14, 15). Different options are possible. A first option is the manual setting of some parameter thresholds per patient (15, 16). This requires manual screening of previous patient data, and it only works well if the parameters are easily understandable. Simple thresholding approaches are however too simple to grasp the large complexity of the problem. Automated personalization is therefore advised, but it normally requires a lot of patient-specific data in order to find a robust algorithm for a specific patient (17). Often, only a limited amount of accurately annotated patient-specific data is available, hence a lot of complex approaches are not useful for making fully patient-specific classifiers. Heuristic automated algorithms allow a low-complex and fast personalization, but might lead to suboptimal results (9).

**Figure 1:**
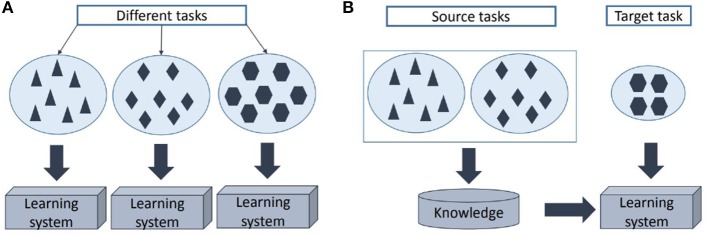
Illustration of the learning procedure using **(A)** traditional machine learning and **(B)** transfer learning.

A more robust and optimal solution can be found through transfer learning (18, 19). In transfer learning, the solution to a classification problem is found by using the solution from a similar problem (“source task”) as a starting point (see Figure 1). This way, less data for the new problem (“target task”) is required in order to get a robust solution as a part of the knowledge is already contained in the reference model. In this paper, a patient-specific heart rate-based seizure detection model is trained through transfer learning by using a patient-independent classifier as a reference model. Therefore, only a limited amount of patient-specific heart rate data is required in order to get a robust patient-specific model, obtained with a relative limited complexity. This paper proposes a new enhanced transfer learning solution that is also able to deal with the large class imbalance and allows to give more importance to seizure samples during classifier training for each individual.

The novelty of this study is threefold. First, a new transfer learning procedure is proposed. An existing transfer learning method is enhanced in order to deal with class imbalance and allows to give increased importance to sensitivity rather than specificity. The latter is a crucial aspect for automated seizure detection. Secondly, the proposed transfer learning approach is applied in order to efficiently personalize heart rate-based seizure detection with a limited amount of patient data. To the best of our knowledge, it is the first time transfer learning is used to personalize automated non-EEG-based seizure detection. Unimodal seizure detection systems based on heart rate typically lead to inaccurate results that are insufficient to be used in practice (20). In this context, the proposed methodology offers a novel solution that can be used as part of a multimodal system, which allows to adapt the detection system to each patient. As a result, a more accurate and usable solution could be achieved (21). These multimodal systems are currently getting close to sufficiently high accuracy for practical usage, but are typically restricted to a certain seizure type (22). The added value of using heart rate in a multimodal system is that it is the ictally most activated modality besides EEG, which allows detection of a wide range of seizure types. Improving the unimodal heart rate-based seizure detection using the proposed method will also improve the multimodal seizure detection, closing the gap to practical usage for a wide range of seizure types. Finally, an in-depth analysis is performed in order to indicate the added value of the proposed transfer learning approach compared to the state-of-the-art literature of heart rate-based seizure detection.

## 2. Materials and Methods

### 2.1. Data Acquisition

The dataset contains recordings from refractory epilepsy patients, who underwent presurgical evaluation at the University Hospitals Leuven (UZ Leuven), Belgium and had at least five FIAS originating from the temporal lobe during the evaluation. The patients were recorded with 10–20 scalp EEG with 1 bipolar ECG channel (lead II) with a sampling frequency of 250 Hz in a fully wired system. The data was recorded continuously and contains both day and night data of the patients within a hospital room. The single-lead ECG signal was continuously unreadable due to noise during 4.6 (patient 13), 8.8 (patient 16), and 7.4 (patient 18) hours. Those segments were removed from the analysis. The remaining data consists of 24 patients with 2,172 h of data. In total, 228 seizures were recorded (see Table 1).

**Table 1:** An overview of the dataset.

	**#**	**RD**					**Mean**	**Range**
**Patient**	**Seizures**	**(h)**	**Hemisphere**	**Origin**	**Age**	**Gender**	**SD (s)**	**SD (s)**
1	10	26	B	T	49	M	31	[24–39]
2	9	63	L	F-T	41	F	13	[13–13]
3	13	71	R	T	27	M	71	[28–96]
4	10	25	B	T	18	M	19	[14–26]
5	11	47	R	T	29	F	50	[40–60]
6	7	148	L	T	26	M	63	[32–116]
7	30	67	R	F-T	19	M	50	[17–90]
8	11	114	L	T	38	M	39	[19–75]
9	8	64	L	T (7), O (1)	28	M	23	[11–31]
10	6	111	EEG not readable		35	M	126	[69–183]
11	6	100	B (5), R (1)	T	67	F	26	[21 31]
12	9	91	R	F-T	24	F	47	[33–85]
13	8	109	R	T	32	M	46	[25–61]
14	5	100	L	T	19	F	56	[29–83]
15	13	110	EEG not readable		49	M	16	[8 30]
16	5	96	L	T	45	M	64	[11–95]
17	7	102	UC (4), R (3)	UC (4), T (3)	18	F	33	[33–33]
18	5	84	L (3), R (2)	T	62	M	125	[89–187]
19	15	113	UC		40	F	23	[6–52]
20	5	113	L	T	41	F	74	[58 83]
21	8	103	L (3), R (5)	T (3), F-T (5)	43	M	67	[29–99]
22	12	115	L (4), R (3), B (5)	T	35	M	27	[17–38]
23	6	101	R	T	23	F	80	[51–108]
24	8	99	R	T (1), O-T (5)	24	F	42	[17–84]
Total	227	2172			[18–67]	14M/10F	50 ± 30	[6–187]

*RD, Recording Duration; SD, Seizure Duration; L, Left; R, Right; B, Bihemispheric; UC, Unclear; T, Temporal; O, Occipital; F, Frontal*.

A clinical expert annotated the seizure onsets and offsets with the use of video-EEG data, without considering the ECG. Afterwards, a neurologist validated the annotations. The seizure duration was defined as the time between EEG seizure onset and offset. However, during 30% of the seizures, the offsets could not be determined. The ethical committee of the UZ Leuven approved the study (approval number S59662). All patients signed the informed consent for their participation in this study.

### 2.2. Preprocessing

Single-lead ECG was used as input for the proposed seizure detection algorithm. First, the heart rate was extracted from the ECG using an approach that used a real-time R peak detection algorithm. It detected the R peaks based on the derivative signal and an adaptive threshold *T*_*t*_ that changed based on the maximal derivatives *P*_*t*_ of the previously detected R peaks (*T*_*t*_ = 0.9 * *T*_*t*−1_ + 0.1 * *P*_*t*_). Next, strong heart rate increases (HRI) caused by sympathetic activations were detected by automated slope analysis on the tachogram. HRI extraction was performed on a filtered tachogram, using a median filter with an order of 15 heart beats. If a heart rate gradient was larger than 1 bpm/s, a strong HRI was assumed. The beginning and end of this HRI was found by analyzing when this heart rate gradient became negative again. The HRI was then assumed to be a strong HRI if thresholds on the length of the HRI (> 8*s*), the achieved peak heart rate during the HRI (>60 bpm) and the (percentual) increase in heart rate during the HRI were exceeded (>10 bpm absolute heart rate increase, >25% percentual increase). These thresholds are based on the findings presented in De Cooman et al. (10).

### 2.3. Feature Extraction

Features were extracted whenever such a strong HRI was detected. In De Cooman et al. (10), it was shown that four features extracted from the HRI led to optimal patient-independent results: the peak heart rate, the heart rate at the start of the HRI, the baseline heart rate (extracted from the minute before the HRI) and the standard deviation of the baseline heart rate period. As the primary goal of this study was to lead to optimal patient-specific results with a limited amount of patient-specific data, only the first two features were used in this study. The reason for this was that most of the performance of the system was already accomplished by those two features. Adding more features to the system requires more training data for robust personalization through transfer learning. Choosing these two features led to an optimal balance between performance and limited requirement of patient-specific data. These features were then classified with either the patient-independent (PI) classifier or the patient-specific (PS) transfer learning classifier.

### 2.4. Patient-Independent Classification

Let ${\left\{x_{i},y_{i}\right\}}_{i=1}^{N}$ be the training data points extracted from patients different than the ones used for testing the algorithm, with ℝ^2^ the data samples and *y*_*i*_ ∈ {−1, +1} the corresponding labels. Let class -1 correspond to seizure samples and class +1 to non-seizure samples. Support vector machines (SVM) will map data points to a higher dimensional space using a (non-)linear transformation φ(*x*), so that the data points can be separated in this space by the hyperplane *w*^*T*^φ(*x*) + *b*, with *w* the unknown weight vector and *b* an unknown constant.

The solution for weighted SVM can be found by solving the following optimization problem

(1) $$\begin{array}{l} \;\underset{w,b,\xi }{\text{min}}\frac{1}{2}||w{||}^{2}+C\sum_{i=1}^{N}c_{i}\xi_{i}\;\\ s.t.\;\{\begin{array}{l} y_{i}(w^{T}\varphi (x_{i})+b)\geq 1-\xi_{i}\\ \xi_{i}\geq 0\;,\forall i\in [1,N] \end{array}\;, \end{array}$$

with ξ_*i*_ the error of the model on *x*_*i*_ and *C* a tunable hyperparameter. A modification of the typical SVM is used here to remove the class imbalance from the dataset (23). The values of *c*_*i*_ are defined as

(2) $$\begin{array}{l} c_{i}=\{\begin{array}{l} \gamma \frac{\left(N^{+}+N^{-}\right)}{2N^{-}}\;:y_{i}=-1\\ \frac{N^{+}+N^{-}}{2N^{+}}\;:y_{i}=+1 \end{array}. \end{array}$$

The parameter γ gives more importance to the correct classification of seizure samples compared to non-seizure samples during classifier training. The Lagrangian of (1) becomes

(3) $$\begin{array}{l} L(w,b)=\frac{1}{2}||w{||}^{2}+C\sum_{i=1}^{N}c_{i}\xi_{i}-\sum_{i=1}^{N}\nu_{i}\xi_{i}\\ \;-\sum_{i=1}^{N}\alpha_{i}\left(y_{i}\left(w^{T}\varphi (x_{i})+b\right)-1+\xi_{i}\right) \end{array}$$

with α_*i*_, ν_*i*_ ≥ 0 the Lagrange multipliers, leading to the dual problem

(4) $$\begin{array}{l} \;\underset{\alpha }{\text{min}}=\frac{1}{2}\sum_{i=1}^{N}\sum_{j=1}^{N}y_{i}y_{j}\alpha_{i}\alpha_{j}K\left(x_{i},x_{j}\right)-\sum_{i=1}^{N}\alpha_{i}\\ s.t.\;\{\begin{array}{ll} \sum_{i=1}^{N}\alpha_{i}y_{i}=0\\ 0\leq \alpha_{i}\leq Cc_{i} & ,\forall i\in [1,N] \end{array}\;, \end{array}$$

with $K\left(x_{i},x_{j}\right)=\varphi {\left(x_{i}\right)}^{T}\varphi \left(x_{j}\right)$the kernel function. The values of the hyperparameter C, γ and the Gaussian kernel parameter σ are taken as reported to be optimal in De Cooman et al. (10). The classifier is trained using the leave-one-patient-out crossvalidation (LOPO-CV) approach, training the classifier on all patients except the one used for evaluating the algorithm.

### 2.5. Personalized Classification Through Transfer Learning

**Figure 2:**
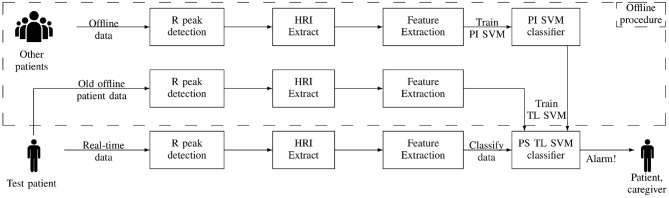
Overview of the proposed transfer learning approach for personalized heart rate-based seizure detection. HRI, heart rate increase; PI SVM, patient-independent support vector machine; PS TL SVM, patient-specific transfer learning support vector machine.

The goal of this study is to personalize the seizure detection classification in order to get an optimal patient performance with a limited amount of patient-specific data. One solution for this is to use transfer learning (TL), which allows to train a new classifier for a problem with a limited amount of data by using a reference classifier that solves a similar problem. The previously trained patient-independent classifier discussed in section 2.4 is used as the reference classifier. This way, the classifier can be personalized with a limited amount of patient-specific data by using the knowledge already incorporated in the patient-independent classifier. An overview of the proposed procedure for personalizing the heart rate-based seizure detection is given in Figure 2.

The newly proposed transfer learning approach is based on the concept described in Yang et al. (24). It states that the weight vector of the new SVM solution should be sufficiently similar compared to the weight vector of the reference SVM solution, while also minimizing the misclassification error on the data of the new problem. In this use case, it means that the weight vector of the new patient-specific and reference patient-independent classifier should be sufficiently similar. The minimization problem proposed in Yang et al. (24) is enhanced here in order to also be able to deal with class imbalance and the increased importance of the sensitivity in seizure detection algorithms. The following minimization problem to create an SVM classifier for the patient-specific data ${\left\{{\tilde{x}}_{k},{\tilde{y}}_{k}\right\}}_{k=1}^{M}$ is proposed

(5) $$\begin{array}{l} \;\underset{{\;}_{\tilde{w},\tilde{b},\tilde{\xi }}}{\text{min}}\frac{1}{2}||\tilde{w}-w{||}^{2}+D\sum_{k=1}^{M}\tilde{c_{k}}\tilde{\xi_{k}}\\ s.t.\;\{\begin{array}{l} \tilde{y_{k}}({\tilde{w}}^{T}\varphi ({\tilde{x}}_{k})+\tilde{b})\geq 1-{\tilde{\xi }}_{k}\\ \begin{array}{l} {\tilde{\xi }}_{k}\geq 0\;,\forall k\in [1,M] \end{array} \end{array} \end{array}$$

with ${\tilde{\xi }}_{k}$ the error of the model on data point ${\tilde{x}}_{k}$ and *w* the weight vector obtained from the patient-independent classifier trained using (1), defined as

(6) $$\begin{array}{l} w=\sum_{i=1}^{N}\alpha_{i}y_{i}\varphi (x_{i}) \end{array}$$

by the original SVM optimization problem. The same transformation function φ and corresponding kernel *K* used in the reference classifier are used here. This minimization problem contains weights ${\tilde{c}}_{k}$ for each ${\tilde{x}}_{k}$, which allows to also take care of the class imbalance and sensitivity importance in the optimization. These weights are defined as

(7) $$\begin{array}{l} {\tilde{c}}_{k}=\{\begin{array}{l} \tilde{\gamma }\frac{\left(M^{+}+M^{-}\right)}{2M^{-}}\;:{\tilde{y}}_{k}=-1\\ \frac{M^{+}+M^{-}}{2M^{+}}\;:{\tilde{y}}_{k}=+1 \end{array}, \end{array}$$

with *M*^+^ and *M*^−^ indicating the number of patient-specific non-seizure and seizure data points. The introduction of these weights ${\tilde{c}}_{k}$ are crucial as they ensure that the personalized classifier is sufficiently stable and targets a sufficiently large sensitivity, which is required for real-life seizure detection systems. Hyperparameter *D* allows to balance between minimizing the errors for the patient-specific data points and minimizing the difference compared to the reference patient-independent classifier (defined by *w*). Parameter $\tilde{\gamma }$ is experimentally initialized to a value of 1.5. The initial value for hyperparameter *D* depends on the amount of seizures available in the training set, and is set to 0.1 for patients with less than 10 seizures and set to 100 for patients with more than 10 seizures. These values are based on the findings reported in De Cooman et al. (25). Nevertheless, these parameters should ideally be optimized per patient, based on their validation performance, but it is very challenging to get robust hyperparameter optimization results due to the low amount of patient training data. Therefore, a more heuristic method is applied, which lowers the values of $\tilde{\gamma }$ (linear decrease of 0.25) and *D* (exponential decrease by factor 1/10) if the resulting classifier leads to 50% more false detections on the patient training data than the available patient-independent approach. This reduction is repeated until the false detection rate (FDR) is dropped below this threshold or minimal values for $\tilde{\gamma }$ (=0.25) and *D* (=0.01) are reached. This procedure is required to avoid that the personalized approach would overtrain on a limited amount of abnormally small ictal heart rate increases, which could lead to a drastic increase in FDR compared to the reference patient-independent classifier.

An optimal solution for (5) is found for the saddle point in the Lagrangian L

(8) $$\begin{array}{l} \underset{\tilde{\alpha },\tilde{\beta }}{\max}\underset{\tilde{w},\tilde{b},\tilde{\xi }}{\min}L\left(\tilde{w},\tilde{b},\tilde{\xi };\tilde{\alpha },\tilde{\beta }\right)\\ \;=\underset{\tilde{\alpha },\tilde{\beta }}{\max}\underset{\tilde{w},\tilde{b},\tilde{\xi }}{\min}\frac{1}{2}||\tilde{w}-w{||}^{2}+D\overset{M}{\underset{k=1}{\sum }}{\tilde{c}}_{k}{\tilde{\xi }}_{k}-\overset{M}{\underset{k=1}{\sum }}{\tilde{\beta }}_{k}{\tilde{\xi }}_{k}\\ \;-\overset{M}{\underset{k=1}{\sum }}{\tilde{\alpha }}_{k}\left({ỹ}_{k}\left({\tilde{w}}^{T}\varphi \left({\tilde{x}}_{k}\right)+\tilde{b}\right)-1+{\tilde{\xi }}_{k}\right) \end{array}$$

with ${\tilde{\alpha }}_{k}$, ${\tilde{\beta }}_{k}\geq 0$ the Lagrangian multipliers. This leads to

(9) $$\begin{array}{l} \{\begin{array}{ll} \frac{\partial L}{\partial \tilde{w}}=0 & \to \tilde{w}=w+\;\sum_{k=1}^{M}{\tilde{\alpha }}_{k}{\tilde{y}}_{k}\varphi ({\tilde{x}}_{k})\\ \frac{\partial L}{\partial \tilde{b}}=0 & \to \sum_{k=1}^{M}{\tilde{y}}_{k}{\tilde{\alpha }}_{k}=0\\ \frac{\partial L}{\partial {\tilde{\xi }}_{k}}=0 & \to {\tilde{\alpha }}_{k}+{\tilde{\beta }}_{k}=D{\tilde{c}}_{k},\forall k\in [1,M] \end{array} \end{array}$$

so that the dual problem of (5) is defined as

(10) $$\begin{array}{l} \underset{\tilde{\alpha }}{\min}\sum_{i=1}^{N}\sum_{k=1}^{M}y_{i}{\tilde{y}}_{k}\alpha_{i}{\tilde{\alpha }}_{k}K\left(x_{i},{\tilde{x}}_{k}\right)-\sum_{k=1}^{M}{\tilde{\alpha }}_{k}+\frac{1}{2}\sum_{k=1}^{M}\sum_{l=1}^{M}{\tilde{y}}_{k}{\tilde{y}}_{l}{\tilde{\alpha }}_{k}{\tilde{\alpha }}_{l}K\left({\tilde{x}}_{k},{\tilde{x}}_{l}\right)\;\\ \;s.t.\;\{\begin{array}{l} \sum_{k=1}^{M}{\tilde{\alpha }}_{k}{\tilde{y}}_{k}=0\\ 0\leq {\tilde{\alpha }}_{k}\leq D{\tilde{c}}_{k}\;,\forall k\in [1,M] \end{array}\text{.} \end{array}$$

Note that

(11) $$\begin{array}{l} \tilde{w}=w+\overset{M}{\underset{k=1}{\sum }}{\tilde{\alpha }}_{k}{ỹ}_{k}\varphi \left({\tilde{x}}_{k}\right) \end{array}$$

indicates that the patient-specific $\tilde{w}$ is a combination of patient-independent and patient-specific information. A new data point ${\tilde{x}}_{n}$ is then classified using

(12) $$\begin{array}{l} y\left({\tilde{x}}_{n}\right)=sign\left(\overset{M}{\underset{k=1}{\sum }}{\tilde{\alpha }}_{k}{ỹ}_{k}K\left({\tilde{x}}_{k},{\tilde{x}}_{n}\right)+\tilde{b}+\overset{N}{\underset{i=1}{\sum }}\alpha_{i}y_{i}K\left(x_{i},{\tilde{x}}_{n}\right)\right)\text{ .} \end{array}$$

The TL classifier is trained and tested using a 5-fold crosstesting scheme, in which 4 folds of patient-specific data are used for training and 1 for testing. This is then repeated 5 times so that each fold is used once as test set.

### 2.6. Alternative Automatic Personalization Solutions

The proposed transfer learning approach is also compared to two different alternatives for personalization. The first alternative includes a fully PS approach which is trained with only PS data using the SVM classifier defined by (1) as in De Cooman et al. (14). The other alternative is a so-called mixed model, in which both PI and PS data are used for training an SVM classifier defined by (1), but adapting the values of *c*_*i*_ in (2) into $c_{i}^{MIX}$:

(13) $$\begin{array}{l} c_{i}^{MIX}\{\begin{array}{c} s_{i}\gamma \frac{N^{+}+N^{-}+M^{+}+M^{-}}{2(N^{-}+M^{-})}:y_{i}=-1\\ s_{i}\frac{N^{+}+N^{-}+M^{+}+M^{-}}{2(N^{+}+M^{+})}:y_{i}=+1 \end{array} \end{array}$$

with

(14) $$\begin{array}{l} s_{i}=\;\{\begin{array}{ll} 4 & :i\in PSdata\\ 1 & :i\notin PSdata \end{array} \end{array}$$

such that misclassification of PS data is more critical during training than misclassification of non-PS data. The value 4 is chosen as recommended in De Cooman et al. (14).

### 2.7. Algorithm Evaluation

In order to compare the different seizure detection algorithms, four performance metrics were used. The first two metrics correspond to the sensitivity (Se, percentage of detected seizures) and false detection rate (FDR, expressed in false positives/hour, FP/h). A seizure is detected if a detection is generated between 1 min prior to the seizure onset and 2 min after the seizure onset. All other detection were classified as false detections. False detections within 1 min of each other were counted as one false detection. In order to combine the Se and FDR in one metric, the F_β_-score with β = 3 is defined as:

(15) $$\begin{array}{l} F_{\beta }=\frac{\left(1+\beta^{2}\right)TP}{\left(1+\beta^{2}\right)TP+\beta^{2}FN+FP} \end{array}$$

with TP, FN, and FP the number of true positives (detected seizures), false negatives (missed seizures) and false positives (false detections). The *F*_3_-score is chosen for this application because it gives more importance to Se compared to FDR. As last metric, the detection delay was determined, which indicates the time difference between the moment of detection and the EEG seizure onset. Average measures over the entire dataset can be expressed as patient average performance (Pat.-Av.), which is the average of the performance of each patient, or overall average (Tot.-Av.), computed on the total number of seizures or recording duration. The first average measure is used, unless specifically mentioned. To prove the significant differences between the algorithms, paired *t*-tests were performed. All results were obtained retrospectively in a simulation which replicated a real-time setting.

### 2.8. Impact of Number of Seizures in Training

One of the advantages of transfer learning is that it allows to train a new classifier with a relative limited amount of data by using a reference classifier (18). In this simulation, the number of required training seizures needed to gain sufficient added value in seizure detection performance was evaluated. Instead of using the full training set, only a few seizures (0–4) from the patient-specific training set were used for training (using the crosstesting scheme described in section 2.5). A model trained with zero seizures is equivalent to a patient-independent model. These training seizures were chosen randomly in 100 simulations for each selected amount of training seizures. This simulation was done for the proposed transfer learning approach and the alternatives for personalization.

### 2.9. Comparison With the Literature

In order to compare the proposed algorithms against the state-of-the-art literature, three algorithms (11, 15, 26) were also implemented and evaluated on the same dataset. The algorithms were implemented based on the corresponding publications. Both Osorio (11) and van Elmpt et al. (15) were based on an algorithm that requires two moving windows: one short window indicating the current heart rate behavior and one longer window indicating the reference/baseline heart rate. Both approaches were described to be patient-independent algorithms, but no preferred threshold values were described in the papers. Therefore, optimal patient-independent threshold values leading to the highest *F*_3_-score were automatically chosen using a LOPO-CV procedure on each training set. The algorithm described in Jeppesen et al. (26) was originally designed to be a patient-specific algorithm (based on non-seizure patient data), but also a patient-independent variation of the algorithm is evaluated here. The used threshold on the parameter value is also optimized in a LOPO-CV procedure.

Also new patient-specific versions of these algorithms from the literature were constructed in an automated fashion. This is done using 5-fold crosstraining, where the threshold values are automatically chosen based on the optimal *F*_3_-score found in the 4 folds of training. Also the test mentioned in section 2.8 is performed for the different state-of-the-art algorithms to compare how fast these can adapt to a patient's characteristics.

## 3. Results

**Figure 3:**
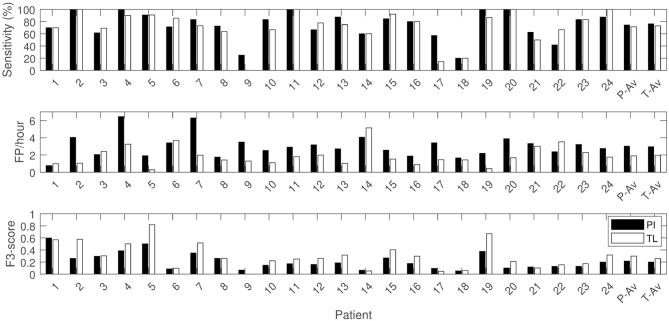
Sensitivity, FDR and *F*_3_-score per patient with patient average (P-Av) and overall average (T-Av) performances for the patient-independent (PI) and transfer learning (TL) algorithm.

The preprocessing procedure discussed in section 2.2 identified 84.7% of the seizures, which gives an indication of the amount of seizures with ictal heart rate increases. Table 2 gives an overview of the results of the patient-independent (PI), fully patient-specific (PS), mixed (MIX) and transfer learning (TL) approaches. The PI algorithm results in an average Se of 75% with 3.0 FP/hour and an *F*_3_-score of 0.22. By adapting the model to the patient characteristics with the TL algorithm, a similar Se is observed (71%) with 37% less false positives (1.9 FP/hour). The average *F*_3_-score is increased to 0.30. Figure 3 shows the results of the proposed TL approach and the reference PI approach for each patient. It illustrates that personalization allows to strongly increase the performance for most patients.

**Table 2:** Results for the patient-independent (PI), fully patient-specific (PS), mixed (Mix) and transfer learning (TL) approach.

	**Se (%)**		**FDR (FP/h)**		**F**_****3****_-**score**	
	**P-Av**	**T-Av**	**P-Av**	**T-Av**	**P-Av**	**T-Av**
**Proposed method**						
PI	**75 ± 22**	**76**	3.0 ± 1.3	3.0	0.22 ± 0.14	0.20
PS	58 ± 27	59	2.2 ± 1.7	2.3	0.24 ± 0.20	0.19
MIX	72 ± 24	74	2.6 ± 1.5	2.5	0.25 ± 0.17	0.22
TL	71 ± 27	73	**1.9 ± 1.1**	**2.0**	**0.30 ± 0.22**	**0.26**
**Jepessen** (26)						
PI	51 ± 32	52	2.8 ± 1.6	3.0	0.16 ± 0.17	0.13
PS	46 ± 23	49	2.0 ± 1.5	1.9	0.23 ± 0.21	0.18
**Osorio** (11)						
PI	71 ± 25	72	2.8 ± 1.2	3.0	0.22 ± 0.15	0.19
PS	75 ± 23	76	2.3 ± 0.8	2.3	0.27 ± 0.18	0.24
**Vanelmpt** (15)						
PI	59 ± 35	61	3.4 ± 2.4	3.5	0.17 ± 0.15	0.14
PS	76 ± 20	77	4.9 ± 3.4	5.2	0.19 ± 0.17	0.13

*Both patient average (P-Av) ± standard deviation and overall average (T-Av) are shown in the table*.

*Bold values indicate the best result for all proposed algorithms for a specific metric*.

**Figure 4:**
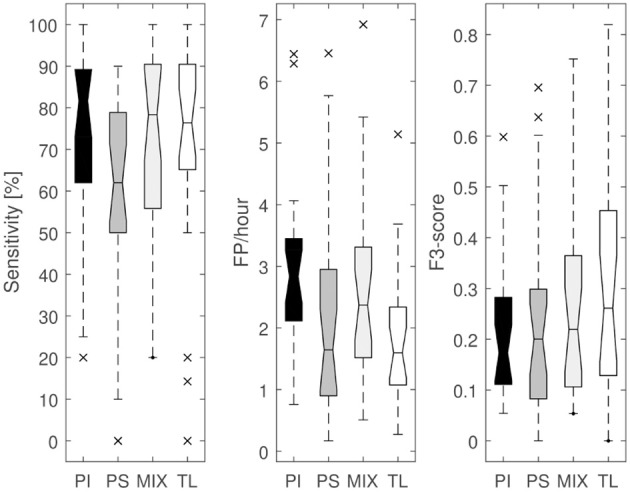
Boxplots of sensitivity, FDR and *F*_3_-score for the patient-independent (PI), fully patient-specific (PS), mixed (MIX) and transfer learning (TL) algorithm.

The alternative mixed approach results in a similar Se, but with on average 0.7 FP/h more than the proposed TL approach. The fully PS approach results in a decreased Se, with a slightly increased FDR (2.2 FP/h) compared to the TL approach. The TL approach did not only result in a decreased average FDR, but also decreased FDR variability over the different patients compared to the personalization alternatives (see Figure 4).

By performing a two-sided paired *t*-test, the sensitivity of the PI and MIX algorithm were found to be not statistically different from the TL algorithm [*p* = 0.29 (PI vs. TL) and *p* = 0.82 (MIX vs. TL)]. However, the FDR of the PI and MIX algorithm are different compared to the TL algorithm [*p* < 0.001 (PI vs. TL), *p*= 0.03 (MIX vs. TL)]. The fully PS approach has a lower Se than the TL algorithm (*p* = 0.01), whereas the FDRs are not significantly different (*p* = 0.29). The *F*_3_-score of the PI, fully PS and MIX algorithm are lower than the TL algorithm [*p* < 0.001 (PI vs. TL), *p* = 0.01 (PS vs. TL), *p* < 0.01 (MIX vs. TL)]. This shows that the proposed transfer learning method is indeed statistically better than the other evaluated approaches for personalization. The average detection delay for the proposed transfer learning approach was 21 s.

Table 2 also shows the results of both the patient-independent and personalized versions of the algorithms from the literature. The algorithms from van Elmpt et al. (15) and Jeppesen et al. (26) result in clearly lower sensitivity with a comparative FDR as the proposed patient-independent approach. The algorithm from Osorio (11) leads to nearly the same average result as the proposed patient-independent approach, with a slightly lower sensitivity, FDR and *F*_3_-score. Both algorithms from Osorio (11) and Jeppesen et al. (26) show an increase in performance by personalizing the thresholds, but result in a lower performance than the proposed TL approach. The personalized version of van Elmpt et al. (15) leads to a strongly increased sensitivity, but also the FDR increases.

**Figure 5:**
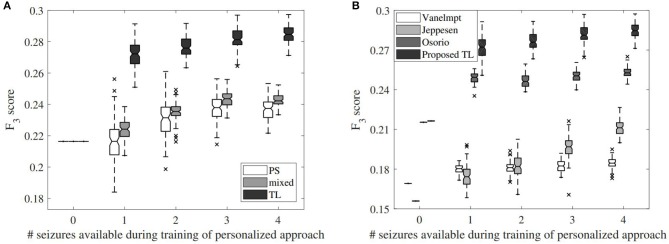
Impact of the number of seizures on the *F*_3_-score performance for different alternatives. **(A)** Effect for the different proposed personalization approaches, including the full patient-specific (PS) approach, mixed approach (mixed) and transfer learning (TL) approach. In case 0 seizures are available for training, the performance of the patient-independent algorithm is shown. **(B)** Effect for the different algorithms implemented from the literature, compared to the proposed transfer learning approach.

As explained in section 2.8, the influence of the number of training seizures on the TL algorithm performance was investigated. Figure 5A shows the impact on the *F*_3_-score performance in function of the number of seizures available during training. The *F*_3_-score for the TL approach strongly improves compared to the PI performance if only one patient-specific seizure is available in the training set. When two seizures are available, the variation between the results decreases, while the average performance increases. The performance further increases by including additional seizures to the training set, while still maintaining a similar variation in results. Figure 5A also shows the effect of the number of seizures in the training set for the other personalization methods, which results in clearly lower performances for each number of available seizures.

The same simulation results for the state-of-the-art algorithms are shown in Figure 5B. The algorithms from Osorio (11) and van Elmpt et al. (15) lead to a decent increase with one available patient-specific seizure, but only increases in accuracy slowly by adding more seizures. This increase is stronger for Jeppesen et al. (26) by adding more seizures, although the results with four available seizures are still worse than the proposed patient-independent algorithm. Although the algorithm from Osorio (11) had a similar patient-independent *F*_3_-score, the proposed personalized TL approach outperforms the personalized version of Osorio (11).

## 4. Discussion

### 4.1. Performance Comparison of the PI and TL Approach

**Figure 6:**
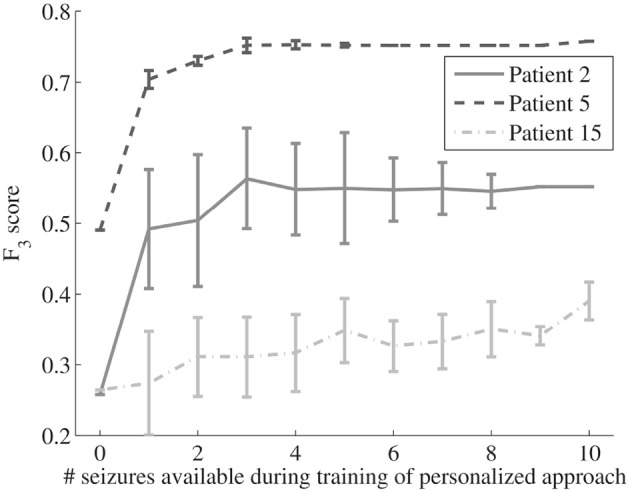
Impact of the number of seizures on the average *F*_3_-score performance (including standard deviations for the performed simulation test) for the proposed personalized transfer learning approach for patients 2, 5, and 15.

Table 2 and Figure 4 show that the mean and median sensitivity of the PI and TL approaches are similar, whereas the FDR decreases and the *F*_3_-score increases. By looking at the patients individually (Figure 3), it can be observed that the TL approach clearly reduced the FDR for some patients without decreasing the sensitivity (e.g., patients 2, 5, and 20). The transfer learning model adapts well to the patient-specific heart rate characteristics. In these patients, the ictal heart rate changes are often very stereotypical, showing little intra-patient variability, which leads to a strong decrease in FDR. Figure 6 illustrates the impact of the number of seizures on the performance for some patients. For patient 5, the personalized approach already gets most performance increase by only including 1 patient-specific seizure. There is also limited variability in the results of the different simulations, showing that the model is accurate and robust for this patient, and little intra-patient variability is found in the ictal HRIs. For patient 2, also a fast personalization can be obtained, but more variability is found between the results of the simulations. This is due to a larger intra-patient ictal variability, so that the selection of seizures in training has a bigger impact on the performance. This impact however reduces if more patient-specific seizures are added to the model, leading to a more robust personalized model. Patient 15 had a lot of seizures during the recording, but these seizures showed a large intra-patient variability in terms of ictal HRIs. This intra-patient variation was mostly caused by the variation of seizure duration, which were typically to short to be strongly differentiable from non-epileptic HRIs. The ictal HRIs are also harder to differentiate from non-ictal HRIs compared to patients 2 and 5, leading to a slower learning curve than those from patients 2 and 5. In general, the steepness of the learning curve and amount of seizures before convergence depend on both ictal and inter-ictal heart rate behavior of each patient.

**Figure 7:**
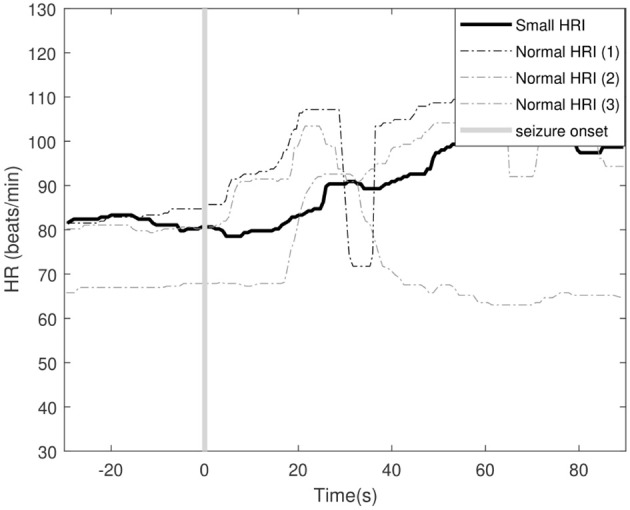
Example of an ictal small heart rate increase (HRI) and 3 normal ictal heart rate increases for patient 8. The vertical line indicates the seizure onset of the different seizures. The shown tachogram signals are filtered using the median filter discussed in section 2.2.

For some patients, however, the sensitivity dropped slightly. This is due to the fact that the model adapts to the patient characteristics. However, the heart rate characteristics during some seizures were atypical (different from other seizures) for that patient. Less severe seizures were typically accompanied with smaller heart rate increases. Those seizures were sometimes not detected with the TL model. An example of a smaller ictal heart rate increase compared to normal ictal heart rate behavior for a particular patient is illustrated in Figure 7. The proposed TL approach adapts to the majority of seizures, and therefore might lead to a missed detection of these atypical seizures. However, this small decrease in sensitivity is often accompanied with a strong decrease in FDR for those patients. The reverse also occurred in some patients, where borderline seizures that were missed by the PI approach are detected by the proposed TL approach (e.g., patient 24). The results above show that, although personalization in general allows to improve the performance, it still has difficulty to counter unpredictable intra-patient variability in seizure behavior. If five seizures within a patient are stereotypical and used for training, an atypical sixth seizure will not be detected accurately using supervised personalization. This is however typically not the case for stronger seizure type (e.g., focal to bilateral seizures), as they are typically easier to detect with heart rate-based seizure detection, even if the algorithm is not specifically trained for this seizure type.

This increase in performance was, however, not achieved for all patients, for example in patient 1. For those patients, rather atypical ictal HR increases are observed, or the HRIs are too weak in magnitude, making it difficult to differentiate them from non-epileptic heart rate activity. If the seizure activity is too similar to non-seizure activity for that patient, the model cannot be improved by means of personalization. Other ECG-based features or features from other modalities might have to be included then in order to achieve better performance for these patients.

Only 84.7% of seizures had ictal HRIs in the analyzed dataset, which is a similar percentage as in the literature (6–8). Personalizing the algorithm will not help to detect these seizures without ictal heart rate changes. Also false detections or missed seizures caused by too strong ECG noise cannot be avoided by the personalization as they can occur both ictally and inter-ictally (27). Other approaches should be used to further improve the performance, such as improved noise removal techniques.

**Figure 8:**
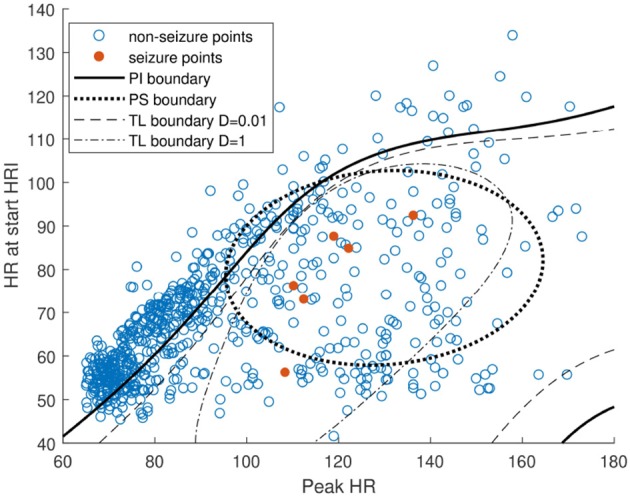
Visualization of SVM boundary of the patient-independent (PI), fully patient-specific (PS), and personalized transfer learning (TL) classifier for different *D*-values (0.01 and 1).

An example of the obtained classifier boundaries for patient 11 are illustrated in Figure 8. The seizure and non-seizure data points are shown together with the PI, PS and TL boundary for two different values of hyperparameter *D* (0.01 and 1), with a fixed $\tilde{\gamma }$ value of 1.5. The PI boundary gives a good general indication, but lacks adaptation to the patient characteristics. The TL approach adapts to these characteristics with a limited amount of available patient-specific data. By choosing a low value for *D* (i.e., 0.01) the TL model will be similar to the PI model because a relative low weight is given to the errors obtained for the patient-specific data compared to the first term in (5), which quantifies how different the new model is to the original one. With a higher value of *D* the model adapts more to the patient-specific data (and the corresponding errors ${\tilde{\xi }}_{k}$) and shows less similarity to the PI boundary. The PS boundary is less optimal, compared to the TL solution. It can be seen from the TL boundary that it still contains information gathered in the PI classifier (especially for low values of *D*), indicating the added value of this approach. This way, the FDR is strongly decreased, without affecting the sensitivity.

### 4.2. Comparison of Alternative Personalization Solutions

Different alternatives for personalization were implemented and evaluated in order to compare with the proposed transfer learning approach (see Table 2 and Figure 4). The naive approach only uses patient-specific data points for training a normal SVM classifier. Despite all patients had at least 5 seizures, this was still an insufficient amount of data for most patients in order to get a robust patient-specific classifier. In Cogan et al. (17), it was mentioned that at least 6–8 seizures were required in order to make a personal algorithm, and the reason for this was to better include the inter-seizure variability of autonomic changes within a patient. Due to the relative low amount of patient-specific data, it often occurred that the classifier was overtrained on a limited amount of seizure data, not taking into account potential fluctuations between different seizures from a patient. This led to a strong decrease in sensitivity, although the FDR was not so much higher than the proposed TL approach. The TL approach is able to better take this inter-seizure variation into account by holding on to the knowledge described by the reference PI classifier.

The mixed model (MIX) uses a mixture of patient-specific data with data from other patients in the training set of a standard SVM training procedure. It produced more robust results than the fully patient-specific approach without strong negative outliers. However, it generated, on average, around 0.5 FP/h more than the proposed TL approach. Applying transfer learning to the reference classifier allows to better take over the information of the PI classifier and translate it to the patient-specific model, whereas the mixed approach would try to create a new model without this prior model knowledge. The proposed transfer learning method not only leads to a better performance, but is also trained faster with an optimization problem which contains less data to analyze.

### 4.3. Impact of Number of Seizures on Personalization

Transfer learning allows to train a personalized classifier for heart rate-based seizure detection. It is however important to have an idea of how much data is actually needed for this. In seizure detection, the amount of seizures is often the restricting factor as some patients have a low seizure frequency. In section 2.8, a simulation study was described to evaluate the impact of the number of seizures in training on the personalized performance (see Figure 5).

The proposed TL approach already leads to a strong increase in *F*_3_ performance by only including 1 seizure in the training set. This shows that with only 1 seizure, the algorithm can already be personalized. There is a lot of variation in the results, which is caused by the heterogeneity of the seizures in the training set. There is also a lot of variation in ictal heart rate changes between different seizures within a patient, so if an atypical seizure is used in training, this will lead to suboptimal results for that patient. By using 2 seizures during training, the *F*_3_ performance increases slightly, and also the variability between results of different simulations decreases. However, from 3 seizures onwards, the variability is no longer strongly decreased with the proposed method. The average performance increases further up until 4 available seizures, and is expected to increase further if more seizures are included in training (17). From a certain amount of seizures, it is however expected that the fully PS approach would lead to a better performance than the proposed TL method. Nevertheless, it is currently not known how many seizures are required for this with the proposed procedure.

Figure 5 also shows the effect of the number of seizures on the alternative personalization approaches. It can be seen that by only having 1 patient-specific seizure in the training data, the TL performance strongly increased, whereas this increase is less evident for the other approaches. For the PS approach, the median performance is increased, but a large portion of the results were actually worse than for the reference PI approach. This is due to the fact that the PS method is often strongly overfitting on data from 1 seizure, which is not a robust way for training a classifier. The average *F*_3_ scores for the fully PS approach increase by adding more seizures, and the variation on performance decreases. The results however remain lower than those from the proposed TL approach when using 4 seizures. The mixed model is more robust than the fully PS approach for a limited amount of seizures, but it slightly loses its added value when more seizures are added to the training set.

### 4.4. Comparison to the Literature

Different algorithms from the literature were also implemented both patient-independently and patient-specifically and evaluated on the dataset described in section 2.1. Table 2 shows that the patient-independent version of the proposed algorithm clearly outperforms the algorithms from van Elmpt et al. (15) and Jeppesen et al. (26), but has a similar result as Osorio (11). A simplified patient-independent algorithm from De Cooman et al. (10) is used here as described in section 2.3, which has shown to outperform the literature. Due to the simplification, the added value of this algorithm over (11) is reduced, but its added value compared to van Elmpt et al. (15) and Jeppesen et al. (26) remained similar.

However, when patient-specific alternatives for these three algorithms were made using the automatic procedure described in section 2.9, the proposed personalization procedure clearly outperforms all these algorithms (see Figure 5B and Table 2). The algorithm from Osorio (11) shows a big increase by personalizing the algorithm, even for only one available seizure, but the added value is much smaller compared to the proposed transfer learning approach. Smaller increase in performance is found in the algorithm of van Elmpt et al. (15), with only a very slow learning curve with increasing number of annotated seizures per patient. A much steeper learning curve is found for the algorithm from Jeppesen et al. (26). Although the algorithm has the lowest patient-independent performance, the performance increases very fast with increasing number of seizures. The algorithm was originally meant for patient-specific evaluation. However, even with four seizures available per patient, it performs worse than the proposed personalized transfer learning method. The proposed personalization method using transfer learning thus not only performs more accurately in general, it also allows a much faster training if only a limited amount of patient-specific seizures are available. This is crucial in practice as some patients have a very low seizure frequency, and thus the personalized algorithm can reach much faster a desired level of accuracy than state-of-the-art algorithms.

### 4.5. Limitations of the Study

There are however some limitations to the performed study that have to be taken into account. First of all, the data is recorded in the hospital, where the patients were restricted to move within their room. This leads to a limited activity of the patient, which can lead to an underestimation of the amount of false detections in practice. However, it is compared to state-of-the-art algorithms from the literature on the same dataset, and has proven to outperform these on this dataset. However, currently no study in the literature has shown results of such heart rate-based algorithms for full day-and-night monitoring in a real home environment.

Furthermore, during the presurgical evaluation, drug treatment can be altered or completely removed. This can influence the results in two ways. First, certain drugs can alter the heart rate variability of the patient, which might lead to different (stronger) ictal and inter-ictal heart rate changes during the presurgical evaluation compared to the home situation. In some patients, indeed small changes in heart rate variability could be found during the first and last day of monitoring. However, the ictal heart rate changes between the first and last day of monitoring were found to be limited compared to the variability that has to be taken into account for the circadian fluctuation of the heart rate features. Therefore, this did not lead to extra missed seizures or a large increase in false detection rate in this study. A second influence is the fact that stronger seizures can occur during the presurgical evaluation compared to the typical home situation. However, only a couple of focal to bilateral seizures were found in our dataset, and the large majority of seizures were perceived to be typical seizures for that patient in their home environment. Other drugs not applied in this study could however have stronger influence on the heart rate variability and the ictal changes. This should be further investigated in future work, as it could influence the usability of data from the presurgical evaluation as training data for such algorithms when used in a home environment.

No dedicated wearable ECG derived device is used in this study. Currently, a large portion of false detections and a small percentage of missed seizures is caused due to poor ECG quality, largely due to the wiring. A previous study has shown that better performance can be obtained by using a wearable ECG device rather than the standard wired hospital ECG (27). This would however be the case for all evaluated algorithms in this study, including those from the literature tested in this study on the discussed dataset.

### 4.6. General Discussion and Future Work

The proposed method allows a fully automated personalization of heart rate-based seizure detection. In literature, personalization was often reached after adjusting thresholds manually after visual inspection of the data (15, 16). This is however a very costly and non-scalable solution. In Jeppesen et al. (26), the personalization was done automatically by adjusting the threshold per patient based on data from a non-epileptic segment of 30 min. Although this is a more scalable solution, only 30 min of non-seizure data does not contain sufficient information to grasp the full complexity of the heart rate variability of that patient (e.g., the circadian rhythm). The proposed solution does take into account much more complexity in a fully automated way, which allows a better implementation in practice.

Despite the increased performance caused by personalization through transfer learning, still a too high FDR is obtained for usage in practice. Personalization allows to solve some of the issues leading to a too high FDR, but is not able to solve all issues related to heart rate-based seizure detection. Some seizures do not contain ictal heart rate changes, and still a large portion of non-epileptic heart rate changes cannot be differentiated from epileptic HRIs with the current techniques. The proposed method should, however, be used as part of a multimodal algorithm, where it is combined with another modality. Such multimodal combination has shown to lead to FDR decreases with factors 5–10 compared to unimodal performance (21). Similar to the seizure detection algorithms, also the used modalities should be chosen for each individual patient based on its typical ictal changes and seizure type. Accelerometers and EMG sensors could lead to an increased performance for the detection of motor seizures (21, 28). For the detection of non-motor focal seizures, behind-the-ear EEG could be used (29). The advantage of heart rate-based seizure detection over other modalities is that ECG is often monitored as well during video-EEG monitoring in the hospital, which allows to get accurately annotated heart rate data to personalize the algorithm. It is also ictally the most activated modality (apart from full EEG), so it is ideal to increase the detection performance of a wide range of seizure types. If a late integration approach is used for combining information from different modalities, the proposed personalized method can be easily integrated and further improve the multimodal performance with a similar accuracy increase as in an unimodal setting.

The proposed transfer learning approach is a supervised approach, which means that annotated data was required. In practice, these annotations can be made in the hospital during, for example, presurgical evaluation, but they could also be made by the patient or their caregivers/family. Extra procedures should then be added to avoid a too big impact of incorrectly annotated data as patients are not always aware about whether they actually had a seizure or not (3, 14). Ideally, an unsupervised approach could be used (9, 25, 26), which indicates that epileptic heart rate activity can be seen as an outlier to normal heart rate activity. This is however only the case during the night (30) or in certain severe seizure types, which makes this approach only sufficiently successful for nocturnal monitoring of severe seizures. Supervised approaches are thus still required for personalizing full day monitoring applications. Due to the supervised approach, still at least one patient-specific seizure is required in order to adapt to the patient characteristics. In patients who have a very low seizure frequency, this might still be a problem for a fast personalization. For these patients, it is then advised to have a large pool of patients, and seizures from patients with similar HRV parameters as the test patient. Then, these seizures could be added to the patient-specific data in order to be able to apply this method.

## 5. Conclusion

Transfer learning allows to personalize heart rate-based seizure detection in a fast and robust way by using only a limited amount of annotated patient-specific data. The false detection rate dropped by 37% compared to the patient-independent approach while maintaining a similar sensitivity. The novel automated personalization approach proposed in this study outperforms the state-of-the-art patient-independent algorithms while also being less prone to overfitting than manual state-of-the-art patient-specific approaches. The proposed method can be used as part of a multimodal algorithm in order to increase the performance and make real-time epileptic seizure warning systems clinically feasible.

## Data Availability Statement

The datasets for this study will not be made publicly available because the patients and healthy participants in the study have not given their approval for the data to be made public. Requests to access the dataset can be sent to the corresponding author.

## Ethics Statement

This study was carried out in accordance with the recommendations of the International Conference on Harmonization guidelines on Good Clinical Practice with written informed consent from all subjects. All subjects gave written informed consent in accordance with the Declaration of Helsinki. The protocol was approved by the Medical Ethics Committee of the University Hospitals KU Leuven.

## Author Contributions

TD, KV, CV, BH, EC, WV, and SV designed the experiments, wrote and revised the manuscript. WV and EC collected the data for the experiments. TD and KV carried out all experiments.

### Conflict of Interest

The authors declare that the research was conducted in the absence of any commercial or financial relationships that could be construed as a potential conflict of interest.
